# Incidence of Second Primary Malignancies Following Colorectal Cancer: A Distinct Pattern of Occurrence Between Colon and Rectal Cancers and Association of Co-Morbidity with Second Primary Malignancies in a Population-Based Cohort of 98,876 Patients in Taiwan

**DOI:** 10.1097/MD.0000000000001079

**Published:** 2015-07-02

**Authors:** Yu-Ting Lee, Chia-Jen Liu, Yu-Wen Hu, Chung-Jen Teng, Cheng-Hwai Tzeng, Chiu-Mei Yeh, Tzeng-Ji Chen, Jen-Kou Lin, Chun-Chi Lin, Yuan-Tzu Lan, Huann-Sheng Wang, Shung-Haur Yang, Jeng-Kai Jiang, Wei-Shone Chen, Tzu-Chen Lin, Shih-Ching Chang, Ming-Huang Chen, Hao-Wei Teng, Jin-Hwang Liu, Chueh-Chuan Yen

**Affiliations:** From the Division of Hematology and Oncology, Department of Medicine, Taipei Veterans General Hospital, Taipei, Taiwan (Y-TL, C-JL, C-HT, M-HC, H-WT, C-CY); National Yang-Ming University School of Medicine, Taipei, Taiwan (Y-TL, C-JL, , Y-WH, C-JT, C-HT, T-JC, J-KL, C-CL, Y-TL, H-SW, S-HY, J-KJ, W-SC, T-CL, S-CC, M-HC, H-WT, J-HL, C-CY); Department of Oncology, Taipei Veterans General Hospital, Taipei, Taiwan (Y-WH, J-HL); Division of Oncology and Hematology, Department of Medicine, Far Eastern Memorial Hospital, New Taipei City, Taiwan (C-JT); Department of Family Medicine, Taipei Veterans General Hospital, Taipei, Taiwan (C-MY, T-JC); and Division of Colon and Rectal Surgery, Department of Surgery, Taipei Veterans General Hospital, Taipei, Taiwan (J-KL, C-CL, Y-TL, H-SW, S-HY, J-KJ, W-SC, T-CL, S-CC).

## Abstract

The purpose of this study is to determine the features of second primary malignancies (SPMs) among patients with prior colorectal cancer (CRC) using a nationwide population-based dataset.

Patients with CRC newly diagnosed between 1996 and 2011, and >1 year of follow-up were recruited from the Taiwan National Health Insurance database. Standardized incidence ratios (SIRs) of SPMs in patients with CRC were calculated.

During the 16-year study period, 4259 SPMs developed among 98,876 CRC patients. The median duration of follow-up was 4.03 years. The SIR for all SPMs was 1.13 (95% confidence interval = 1.10–1.17). Compared with the general population, a higher incidence of thyroid, prostate, ovarian, and hematologic malignancies developed among patients with colon cancer, whereas the risk for bone and soft tissue cancers increased among patients with rectal cancer. The risk for breast, bladder, kidney, lung, and uterine cancers was significantly higher in patients with colon and rectal cancers than the general population. The risk for liver and biliary tract cancers declined in patients with rectal cancer. Based on multivariate analysis among patients with CRC, age ≥70 years, men, chronic obstructive pulmonary disease (COPD), cirrhosis, and dyslipidemia were independent predictors of an SPM.

In conclusion, patients with CRC were at increased risk for a second cancer. The pattern of SPMs was distinct between patients with colon and rectal cancer. Age, men, COPD, cirrhosis, and dyslipidemia were independent risk factors for SPMs. Surveillance and education should be provided for survivors with respect to risk for SPMs.

## INTRODUCTION

Colorectal cancer (CRC) is one of the most common cancers in Taiwan. Large-scale screening for CRC has reduced the mortality rate through early detection and treatment.^1–3^ In addition, the overall survival of patients with advanced CRC was significantly improved with treatments provided by multidisciplinary teams.^4^ Therefore, the late outcomes of CRC survivors, including the increased risk of second primary malignancies (SPMs),^5^ become more and more important in clinical practice.

SPMs are a major issue with respect to the long-term sequelae of cancer survivors. SPMs can be associated with patient genetic backgrounds, cancer-related treatment, lifestyle, and environmental risk factors.^6^ SPMs have been reported in different types of cancer. Several publications have reported SPMs in CRC patients, most of which involved Western or other developed countries.^7–10^ There have been few large-scale population-based studies of SPMs from Asian countries.^8^ Most reports have demonstrated that CRC survivors face a higher cancer standardized incidence ratio (SIR),^7–11^ but few studies have accounted for racial disparities.^12^ Most studies have obtained data from national cancer registries, which limit the potential to consider underlying comorbidities.

Taiwan's National Health Insurance Research Database (NHIRD) provides nationwide data for health research. All patients with malignant diseases have catastrophic illness certifications, substantially reducing their medical expenses. These features make the NHIRD an ideal tool for the analysis of cancer risk. Using the NHIRD, we compared the overall incidence of malignancies and the incidence of specific types of SPMs among CRC patients with the expected incidence in an age-, sex-, and calendar year-matched general population. In addition, the present study investigated the link between comorbidities and SPMs.

## PATIENTS AND METHODS

### Data Sources

Taiwan's National Health Insurance (NHI) is a compulsory health insurance program. Established in 1995, NHI covers the comprehensive medical care of all Taiwanese residents; the coverage rate is >99%.^13^ NHI covers inpatient, outpatient, emergency, and traditional Chinese medical services. The NHIRD provides nationwide population-based data for healthcare research. The catastrophic illness registry incorporates several NHI databases, including NHI claims data, enrollment files, and drug prescription registry, to offer comprehensive patient characteristics and utilization of healthcare resources of all patients enrolled in the catastrophic illness registry. Patients under NHI's catastrophic illness registry are given copayment exemption.

All NHIRD data that might possibly identify a particular patient is encrypted. As required by the policies of Taiwan's National Health Research Institutes and the NHI Administration, all data are confidential. The study has been approved by Taipei Veterans General Hospital's Institutional Review Board (No. 2013-10-002CE).

### Study Population

We conducted a retrospective cohort study between January 1, 1996 and December 31, 2011. Patients with newly diagnosed CRC (pathologically confirmed) were retrieved via the Registry of Catastrophic Illness according to the International Classification of Diseases, Ninth Revision, Clinical Modification (ICD-9-CM) major codes 153 and 154. SPMs were defined as those malignancies that occurred 1 year after diagnosis of CRC. Therefore, participants who were followed <1 year, or SPMs diagnosed within the first year after diagnosis of CRC were not enrolled. Because it is difficult to distinguish between synchronous and metachronous CRCs, second primary CRCs were not analyzed. We also excluded neoplasms of the small intestine to avoid misclassification. Information on diagnosis age, sex, and comorbidities, including autoimmune diseases, chronic obstructive pulmonary disease (COPD), diabetes mellitus (DM), dyslipidemia, end-stage renal disease (ESRD), and liver cirrhosis, was collected from the NHIRD.

### Statistical Analysis

SPM development was the main dependent variable. We used the Registry for Catastrophic Illness Patients (RCIP), which requires pathological confirmation to identify subjects diagnosed with cancer. Those diagnosed with CRC were followed until the following: SPM development, dropout from the NHI program, death, or the 2011 year-end.

We used SIRs, which means the ratio of the observed number to the expected number of cancer incidents, to determine the SPM risk among this CRC cohort. We determined the expected cancer numbers by multiplying the national cancer incidence according to age in 5-year intervals, sex, and calendar year by the specific stratified person-time variable accrued from the CRC cohort. Cancer incidence among the general population was retrieved from the Taiwan National Cancer Registry, and the 95% confidence intervals (CIs) for SIRs were calculated by using a Poisson probability distribution. Cases were separated based on their anatomic primary site—that is, colon cancer and rectal cancer—and SIRs were calculated for each SPM type in the colon and rectal cancer groups. Additionally, SIRs were determined for subgroups by sex and age; in order to avoid surveillance bias and to analyze any time effects on SPM, subgroup analysis was also stratified by time, beginning from CRC diagnosis.

We used univariate and multivariate Cox proportional hazards models to identify predictors of SPM occurrences among CRC patients. All factors in the univariate analysis with a *P* < 0.1 were included in the multivariate analysis.

The Perl programming language (version 5.12.2) was used for extracting and computing data, the Microsoft SQL Server 2012 (Microsoft Corp., Redmond, WA) for data linkage and processing, and SAS 9.2 software (SAS Institute Inc., Cary, NC) for all statistical analysis. A *P* value < 0.05 is considered statistically significant.

## RESULTS

### Characteristics of the Study Population

According to our criteria, we identified 98,876 patients with CRC in the catastrophic illness registry of the NHIRD, including 55,729 (56.4%) men and 43,147 (43.6%) women, with a median age of 67 years at the time of diagnosis (interquartile range, 56–75 years). Overall, the cohort was observed for 520,175 person-years between 1996 and 2011. The median follow-up time was 4.03 years (interquartile range, 2.14–7.49 years). The demographic data of this cohort are presented in Table 1.

**TABLE 1:**
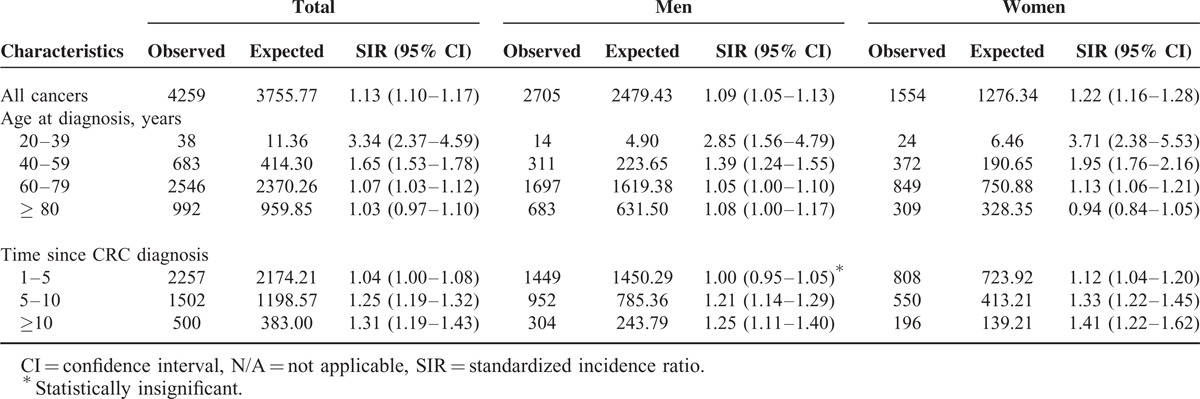
Standardized Incidence Ratios According to Gender, Age at Diagnosis and Duration of Colorectal Cancer

### All Cancers

During the observation period, 4259 patients developed SPMs. Compared with the general population, CRC patients had a significantly increased risk of all cancers (SIR = 1.13, 95% CI = 1.10–1.17). The SIR was 1.09 (95% CI = 1.05–1.13) for men and 1.22 (95% CI = 1.16–1.28) for women. The median time of SPM development was 4.7 (2.7–7.5) years.

The age-specific SIR was calculated. Patients who were 20–39 years of age at the time of diagnosis had a higher SIR for all cancers (3.34, 95% CI = 2.37–4.59) than other age groups (40–59, 60–79, and ≥ 80 years have SIRs of 1.65 [95% CI = 1.53–1.78), 1.07 [95% CI = 1.03–1.12], and 1.03 [95% CI = 0.97–1.10], respectively). Moreover, the SIR in women ≥ 80 years of age was not significantly higher than that of the general population (0.94, 95% CI = 0.84–1.05). Subgroup analysis according to duration of CRC showed that the SIRs of patients followed-up for 1–5, 5–10, and ≥ 10 years were 1.04 (95% CI = 1.00–1.08), 1.25 (95% CI = 1.19–1.32), and 1.31 (95% CI = 1.19–1.43), respectively. The results of the subanalysis are summarized in Table 2.

**TABLE 2:**
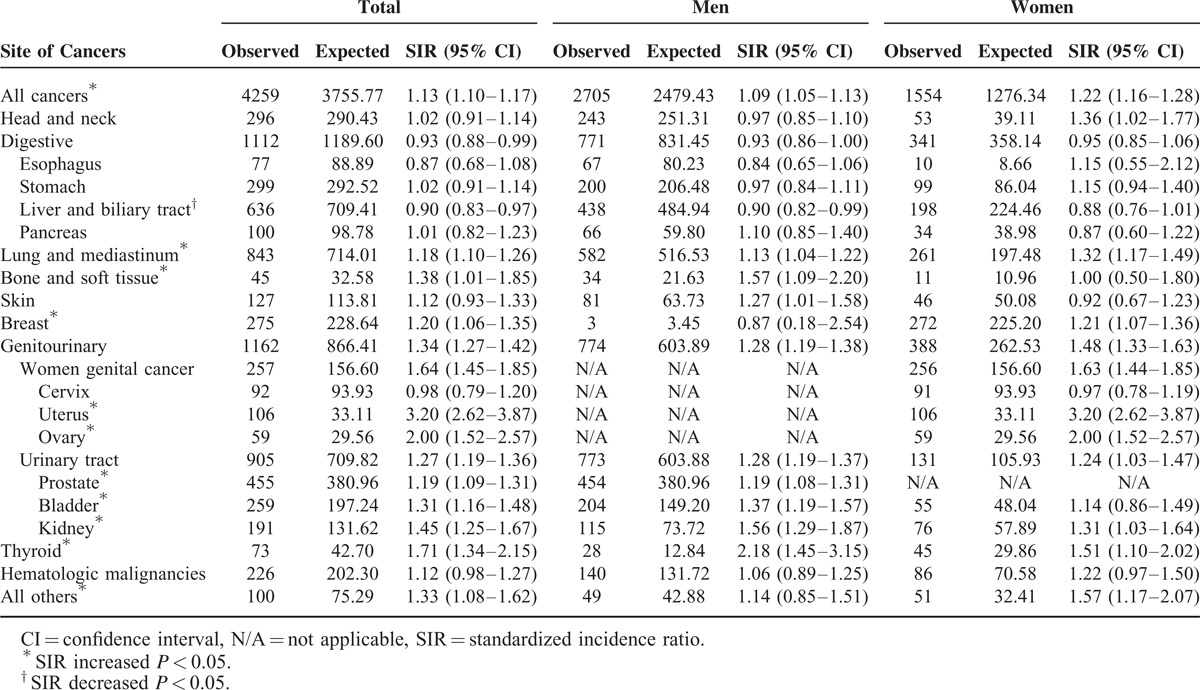
Standardized Incidence Ratios (SIRs) for Specific Cancer Types Among Patients with Colorectal Cancer

### Specific Cancer Types

Patients with CRC were shown to be at significantly increased risks for cancers of the uterus (SIR = 3.20, 95% CI = 2.62–3.87), ovaries (SIR = 2.00, 95% CI = 1.52–2.57), thyroid (SIR = 1.71, 95% CI = 1.34–2.1), kidneys (SIR = 1.45, 95% CI = 1.25–1.67), bones and soft tissues (SIR = 1.38, 95% CI = 1.01–1.85), bladder (SIR = 1.31, 95% CI = 1.16–1.48), breasts (SIR = 1.20, 95% CI = 1.06–1.35), prostate (SIR = 1.19, 95% CI = 1.09–1.31), and lungs and mediastinum (SIR = 1.18, 95% CI = 1.10–1.26). In contrast, the risk for liver and biliary tract cancers was decreased (SIR = 0.90, 95% CI = 0.83–0.97). The incidence of head and neck, esophageal, skin, gastric, and pancreatic cancers, as well as hematologic malignancies, was not significantly different from that in the general population (Table 3).

**TABLE 3:**
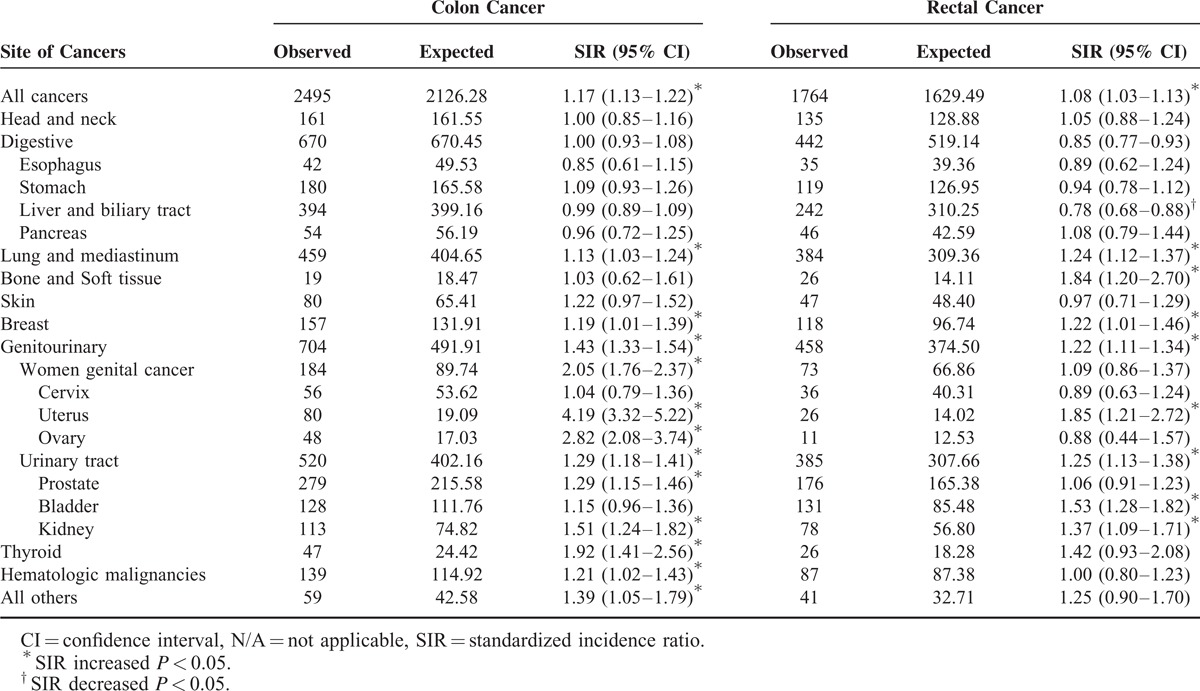
Standardized Incidence Ratios (SIRs) for Specific Cancer Types Among Patients with Colon Cancer and Rectal Cancer, Respectively

### Distinct Patterns Between Colon and Rectal Cancers

Compared with the general population, the incidence of SPMs increased in patients with colon or rectal cancer, and the SIRs were 1.17 (95% CI = 1.13–1.22) and 1.08 (95% CI = 1.03–1.13), respectively. In analyses stratified by the colon and rectal cancer groups, the incidence of thyroid (SIR = 1.92, 95% CI = 1.41–2.56) and prostate cancers (SIR = 1.29, 95% CI = 1.15–1.46), as well as hematologic malignancies (SIR = 1.21, 95% CI = 1.02–1.43), increased among the colon cancer group, whereas subsequent bone and soft tissue cancers occurred more frequently among the rectal cancer group (SIR = 1.84, 95% CI = 1.20–2.70).

As mentioned previously, the risk for liver and biliary tract cancers was decreased in CRC patients. If we consider colon and rectal cancers individually, the decline in the risk for liver and biliary tract cancers was confined to rectal cancer patients (SIR = 0.78, 95% CI = 0.68–0.88). Similar findings were also found when hepatocellular carcinomas (HCCs) and bile duct cancers were analyzed separately. The SIRs for cancers of the breast, uterus, lung and mediastinum, bladder, and kidney were significantly elevated in both groups; the details are listed in Tables 4 and 5.

**TABLE 4:**
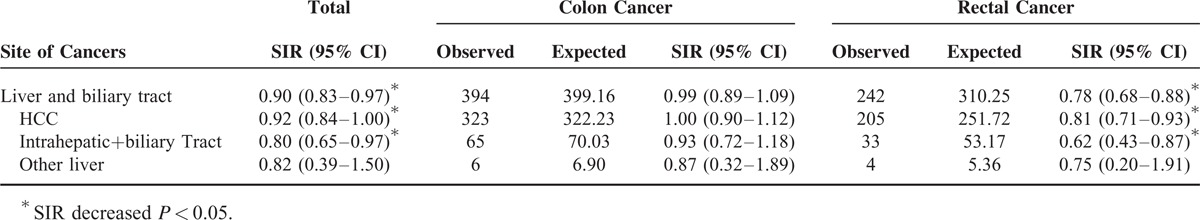
Standardized Incidence Ratios (SIRs) for Specific Histologic Types of Liver and Biliary Tract Cancer Among Patients with Colon Cancer and Rectal Cancer, Respectively

**TABLE 5:**
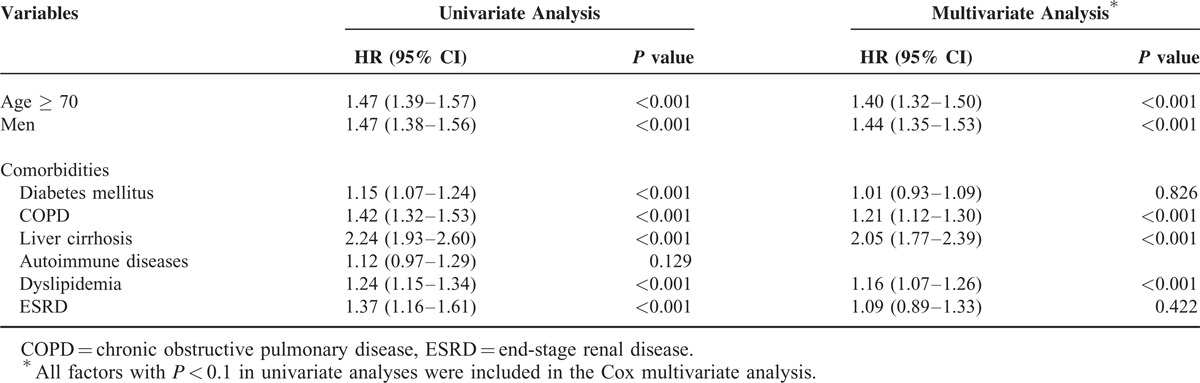
Risk Factors for Cancer Development in Patients with Colorectal Cancer

### Risk Factors for SPMs

A Cox regression model was used to predict the potential risk factors for SPMs. Univariate Cox proportional hazards analysis showed that age ≥ 70 years, men, liver cirrhosis, COPD, ESRD, DM, and dyslipidemia were significantly associated with a higher risk for SPMs. The treatment modality, including surgery and chemotherapy, was not associated with SPMs. Multivariate analysis showed that age ≥ 70 years (hazard ratio [HR] = 1.40, 95% CI = 1.32–1.50; *P* < 0.001), men (HR = 1.44, 95% CI = 1.35–1.53; *P* < 0.001), liver cirrhosis (HR = 2.05, 95% CI = 1.77–2.39; *P* < 0.001), COPD (HR = 1.21, 95% CI = 1.12–1.30; *P* < 0.001), and dyslipidemia (HR = 1.16, 95% CI = 1.07–1.26; *P* = 0.001) remained significant independent predictors of cancer development.

## DISCUSSION

In the present study, we demonstrated that the incidence of SPMs is higher in patients with CRC than in the general population (SIR = 1.13). In addition to the SPMs previously reported,^7,9,10^ we found an increased risk for bone and soft tissue cancers in patients with CRC. Of note, opposite risks for a second liver cancer were observed in subgroup analysis. Indeed, the incidence of second liver cancers decreased among those patients with rectal cancer. We also found that COPD, liver cirrhosis, and dyslipidemia were significantly associated with SPMs based on multivariate analyses. Younger patients with CRC also had a higher cancer SIR.

Taiwan NHI is a mandatory program for all Taiwan's residents. Thus, its referral bias is low and follow-up is comprehensive. In addition, all patients with cancer enrolled in RCIP require histologic confirmation, which makes the diagnosis of cancers both reliable and exhaustive. These features, as well as unbiased subject selection and SIR estimations with matching ages, sex, and calendar years, strengthen the design of this nationwide population-based study and support the validity of our results.

This is the first large population-based study in Asia to evaluate the risk for SPMs among CRC patients. Several prior studies have observed an increased risk for SPMs among patients with CRC. Evans et al reported a link in men diagnosed with CRC before 60 years of age and in women diagnosed with CRC after 65 years of age^7^. A recent study in Australia conducted by Dasgupta et al demonstrated a significant risk of developing non-CRC (SIR = 1.24).^9^ Phipps et al used SEER registries and reported a slight but significantly increased risk of sequential non-CRC (SIR = 1.04).^10^ Among CRC survivors, an increased risk for bladder, kidney, prostate, stomach, lung, ovarian, breast, and thyroid cancers, as well as melanoma, have been well described in previous studies.^7–12,14–17^ The same results were also found in the present study. Additionally, an excess risk for bone and soft tissue cancers was observed in the present study.

Previous studies have shown that CRC from different primary locations may have SPMs with distinct patterns.^9,10^ Phipps et al has shown that the incidence of endometrial cancer is elevated in patients with a proximal colon cancer, but not rectal cancer.^10^ In the present study, we also found an increased incidence of thyroid and prostate cancers, and hematologic malignancies in patients with colon cancer, whereas subsequent bone and soft tissue cancers occurred more frequently among the rectal cancer group. CRC is a heterogeneous disease with different molecular subtypes,^18,19^ and different locations of tumors may have distinct genetic features. Several studies have demonstrated that CRC of the right colon has a higher chance of methylation, microsatellite instability, and genetic mutation than tumors originating from the left colon;^20,21^ however, it is not easy to associate molecular heterogeneity in primary CRC with the risk for SPMs.

In the present study, we demonstrate that the risk for liver and biliary tract cancers declines in patients with CRC, especially in patients with rectal cancer. A similar finding was also observed by Phipps et al.^10^ One possible mechanism to explain the decreased risk for SPMs in patients with CRC is inflammation. A recent study showed that deoxycholic acid (DCA), a secondary bile acid known to cause DNA damage, may return to the liver through enterohepatic circulation and provoke the senescence-associated secretory phenotype in hepatic stellate cells, which in turn secretes pro-inflammatory and tumor-promoting factors in the liver and facilitates tumor development.^22^ Resection of the colon or rectum in patients with CRC may reduce the chance of these carcinogens entering the liver; however, further studies are needed to explore this interesting phenomenon.

Stratified age analysis demonstrated that the relative risk for SPMs in patients with CRC decreased progressively with increasing age at the time of initial diagnosis in earlier studies^9,11^ and the present study. Age is the strongest risk factor for cancer. Since young patients have a relatively low incidence of comorbidities, the estimated risk for SPMs in younger patients with CRC is probably explained by genetic predisposition.

The mechanism of increased risk for SPMs in patients with CRC is uncertain. Some etiologies, including underlying genetic background, lifestyle, and environmental risk factors, are involved with the development of cancer. Patients with hereditary nonpolyposis colorectal cancer (HNPCC) may have a higher risk of extra-colonic cancers.^11^ Taiwan has also reported few cases of familial hereditary CRC,^23,24^ which may influence the pattern of SPMs. Smoking contributes to lung, colorectal, and bladder cancers.^25–27^ In the present study, we showed that COPD, a smoking-related comorbidity, is significantly associated with SPMs in patients with CRC. Phipps et al hypothesized that the increased risk for SPMs after CRC may be high for organs that are developmentally related to endoderm-derived epithelia^10^. These embryologically related tissues may be expected to respond in a similar fashion to environmental exposures and carcinogens, and may be similarly susceptible to aberrant epigenetic changes. Finally, treatment factors are predisposed to the development of sequential cancers. Both radiation and chemotherapy are positively associated with secondary cancers;^28,29^ however, we did not find a significant association of prior treatment with the occurrence of SPMs. Furthermore, our observational study includes only epidemiological results, which need further research into the mechanism.

The present study had several limitations. First, several potential confounders, including tobacco use, alcohol use, obesity, and a family history of malignancy, could not be obtained from the data. It is believed that CRC survivors with obesity have higher risk of a second cancer.^30^ Second, we lacked data on histologic features, genetic factors, stages of cancer, and treatment modality in these patients, making it impossible to assess the correlation between disease severity, treatment modality and incidence of SPMs. Finally, carcinogenesis is a lengthy process; therefore, a longer follow-up period may be necessary to detect certain types of cancers.

In conclusion, this nationwide population-based study shows that CRC patients have an increased risk for SPMs, especially men, those ≥70 years of age, and those with liver cirrhosis, COPD, or dyslipidemia. The incidence of secondary hepatocellular carcinoma was shown to decline among those with rectal cancer. The longer CRC patients were followed, the higher the SIR of SPMs. More intensive surveillance may be cost-effective in these groups of patients, leading to earlier detection and treatment of cancers.
